# Angiotensin converting enzyme defects in shock: implications for future therapy

**DOI:** 10.1186/s13054-018-2202-y

**Published:** 2018-10-28

**Authors:** Lakhmir S. Chawla, Steve Chen, Rinaldo Bellomo, George F. Tidmarsh

**Affiliations:** 1 Veterans Affairs Medical Center, San Diego, CA USA; 2 La Jolla Pharmaceutical Company, 4550 Towne Centre Court, San Diego, CA 92121 USA; 3 School of Medicine, The University of Melbourne, Parkville, Melbourne, VIC Australia; 4 Stanford School of Medicine, Stanford, CA USA

**Keywords:** Angiotensin insufficiency, ACE defect, Vasodilatory, Bradykinin, Vasodilatory shock, Sepsis

## Background

Patients who develop vasodilatory shock, particularly when caused by an inflammatory condition like sepsis or pancreatitis, have evidence of significant endothelial injury as manifested by coagulation disorders and increased capillary permeability [1, 2]. Since angiotensin converting enzyme (ACE) activity is primarily endothelium membrane-bound [3], patients with vasodilatory shock may develop an ACE defect [4, 5]. The pulmonary and renal capillary beds hold the majority of endothelium-bound ACE and patients with acute respiratory distress syndrome (ARDS) have increasing ACE insufficiency with increased severity of lung injury [5, 6]. Moreover, previous studies have demonstrated that endotoxemia causes a decrease in ACE function [7, 8], and, finally, ACE function has been shown to be important in sepsis outcomes [5, 9, 10]. Based on these findings, investigators from the first ATHOS trial have suggested that endothelial dysfunction in vasodilatory shock may cause a significant ACE defect that results in angiotensin II (ANG-2) insufficiency [4].

## Main text

**Fig. 1:**
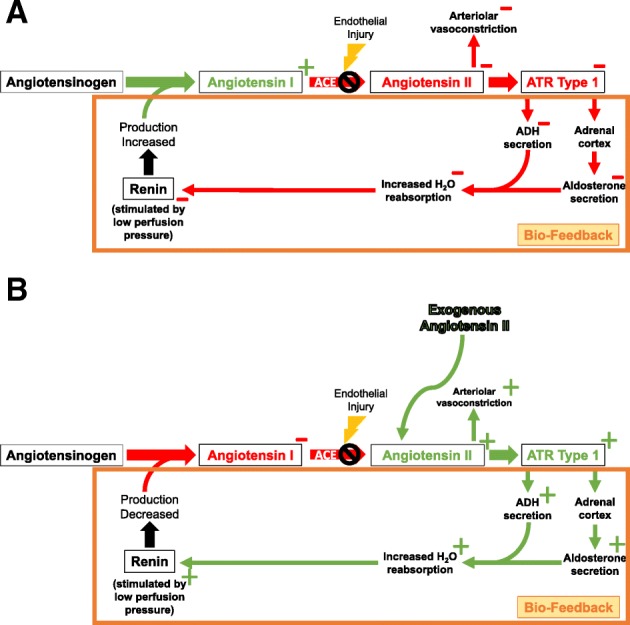
Proposed biofeedback mechanism. **a** Endothelial injury causes disruption of the normal renin-angiotensin-aldosterone system (RAAS) pathway via depleted ACE functionality, resulting in reduction of angiotensin II, increased production of renin, and ultimately increased ACE precursors. **b** With the addition of exogenous angiotensin II, a biofeedback mechanism is triggered via engagement of the angiotensin II type 1 receptor, resulting in increased blood pressure and decreased production of angiotensinogen and/or renin, ultimately reducing angiotensin I levels, *ADH* antidiuretic hormone

In order to test this hypothesis, as part of the ATHOS-3 trial, endogenous ANG-1 and ANG-2 levels were measured prior to study drug infusion at baseline and again 3 h after initiation of exogenous ANG-2 or placebo. One goal of these assessments was to determine if ACE function, as measured by the ANG-1 and ANG-2 levels, was normal. In healthy patients, ANG-2 levels are generally higher than ANG-1 levels [11]. In the ATHOS-3 trial, ANG-1 and ANG-2 levels were significantly elevated with the ANG-1 levels much more elevated than ANG-2 levels, leading to a relative ANG-2 deficiency [12]. This finding is consistent with other studies showing decreased ACE activity in vasodilatory shock and implies that ACE is highly dysregulated in this setting [5, 6]. An unexpected finding was change in ANG-1 at 3 h after baseline. As expected, patients who received placebo did not have a significant change from baseline to 3 h in ANG-1 (median (IQR) values were 238 (75–653) at baseline and 218 (76–553) at 3 h). However, patients who received exogenous ANG-2 demonstrated a significant decrease in ANG-1 levels (the median (IQR) values were 260 (72–679) at baseline and 166 (47–383) at 3 h, *p* < 0.0001). We hypothesize that this rapid decrease in ANG-1 may be mediated by a biofeedback mechanism: exogenous ANG-2 causes engagement of the ANG-2 type 1 receptor, resulting in increased blood pressure and decreased production of angiotensinogen and/or renin (Fig. 1a, b).

**Fig. 2:**
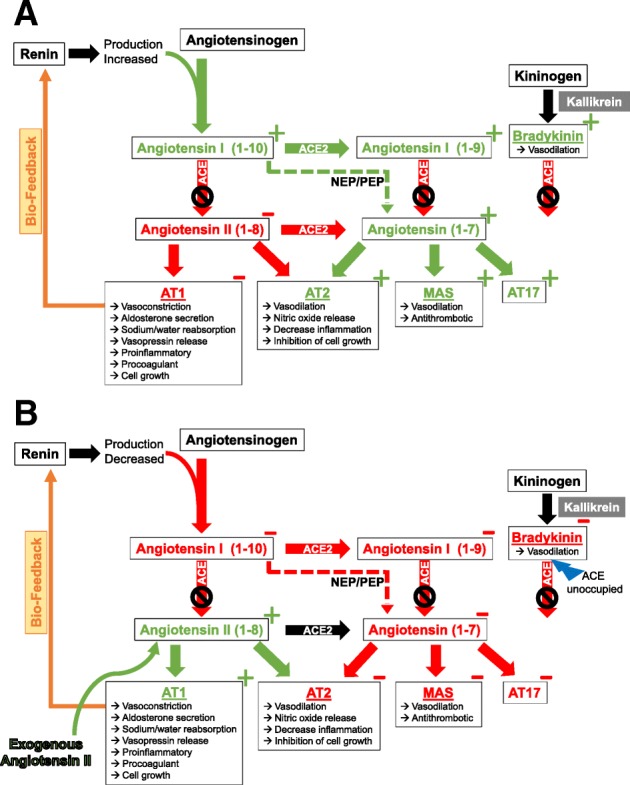
Proposed mechanism of angiotensin metabolism during shock and with the addition of exogenous angiotensin II. **a** When ACE is inhibited by ACE inhibitors or during vasodilatory shock, bradykinin, angiotensin I, and angiotensin 1-7 (ANG 1-7) increase. ANG 1-7 has effects that are the opposite those of angiotensin II. Both ANG 1-7 and bradykinin are vasodilatory, and they may build up when ACE is not functional, compounding the issue of angiotensin II insufficiency. **b** The addition of exogenous angiotensin II provides a direct benefit by ameliorating the angiotensin II insufficiency. However, it may also provide benefit by reducing vasodilatory angiotensins via biofeedback, resulting in ACE availability and bradykinin degradation (*blue lightning bolt*). *NEP* neutral endopeptidase, *PEP* prolyl endopeptidase

Studies of hypertension patients have shown that ACE inhibition causes increases in bradykinin, ANG-1, and other angiotensin peptides such as ANG 1-7 [11]. ANG 1-7 has been shown to cause vasodilation and to decrease blood pressure [13] (Fig. 2a). Similarly, bradykinin, an ACE substrate, has vasodilatory properties [14, 15]. These data suggest that patients with ACE defect and vasodilatory shock may suffer from a simultaneous excess of the vasodilatory mediators normally metabolized by ACE and a lack of ANG-2 generation. The addition of exogenous ANG-2 in this subset of patients may provide a dual benefit by ameliorating the ANG-2 insufficiency, thereby improving blood pressure and reducing vasodilatory angiotensins. The decrease in vasodilatory angiotensins, which are also ACE substrates, may, in turn, improve ACE availability and increase bradykinin degradation (Fig. 2b). This concept is a preliminary hypothesis that will require further investigation. However, if this mechanism can be verified, exogenous ANG-2 therapy may logically provide a therapeutic option for vasodilatory shock patients with ACE defects. Moreover, therapies that utilize agents to decrease vasodilatory ACE substrates like recombinant ACE, ACE-2, or renin inhibitors (i.e., aliskrinin) could be combined with exogenous ANG-2 to further potentiate this therapeutic approach. Similarly, treatment with exogenous ANG-2 may even have the potential to treat ACE inhibitor-associated angioedema by decreasing vasodilatory angiotensins and bradykinin levels.

## Conclusions

Endothelial injury during shock may lead to ACE defects, which in turn may cause an increase in vasodilatory mediators that are normally metabolized by ACE and a relative or absolute decrease in ANG-2. These pathophysiological derangements may be beneficially affected by ANG-2 infusion. This mechanism of action in shock justifies further investigation of ACE activity, bradykinin levels, and ANG 1-7 levels in vasodilatory shock and may be an important target for future therapeutic intervention.

## Abbreviations

ACE: Angiotensin converting enzyme
ANG: Angiotensin
ARDS: Acute respiratory distress syndrome
